# PezT: A Bacterial Suicide Gene

**DOI:** 10.1371/journal.pbio.1001036

**Published:** 2011-03-22

**Authors:** Caitlin Sedwick

**Affiliations:** Freelance Science Writer, San Diego, California, United States of America

**Figure:**
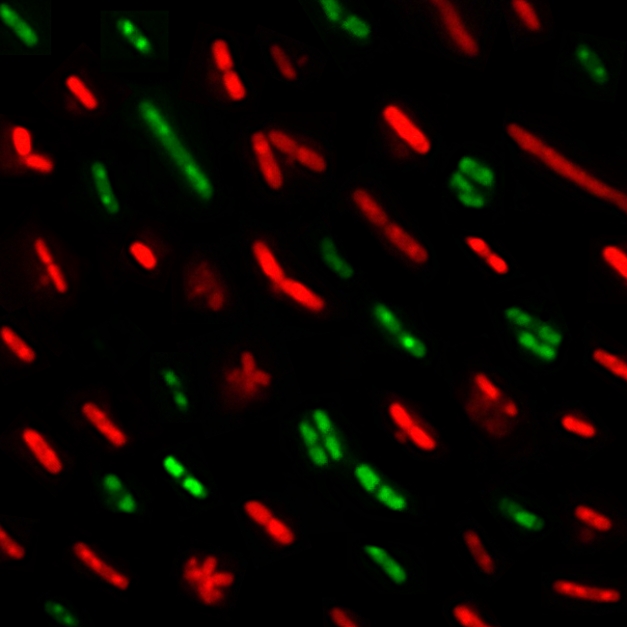
The toxin PezT kills bacterial cells by disrupting cell wall synthesis. Cells exposed to the baneful molecular mechanism of PezT lose the ability to divide (green) until they eventually burst and die (red).

Bacteria are highly skilled at biological warfare. They have a range of weapons at their disposal: everything from secreted toxins to camouflage. Many bacteria can even make use of what's known as a toxin–antitoxin system to program their own individual suicide in support of the population's survival. In this system, a bacterial toxin lies dormant within the bacterium, wrapped up and prevented from becoming active by an antitoxin protein. Under conditions of stress, the antitoxin either stops being produced or is degraded, freeing the toxin to perform its attacks. One such toxin–antitoxin system, PezAT (also known as the epsilon/zeta system), is expressed in many pathogenic bacteria, but its mechanism of action is not known. In this issue of *PLoS Biology*, Hannes Mutschler, Anton Meinhart, and colleagues explain how this toxin–antitoxin system operates.

The PezAT system has been difficult to study because the toxin half of the system, PezT, is deadly when expressed in cells without its antitoxin counterpart, PezA. However, Meinhart and his team observed that a mutant version of PezT—one lacking the last 11 amino acids of its C-terminus—still kills the bacteria that host it, but does so slowly enough to allow its mechanism of killing to be studied. Because truncated PezT kills slowly, the researchers had time to observe that it does so by distorting the shape of the bacteria and ultimately bursting the cells. These observations suggested that it acts by impairing the integrity of the bacterial cell wall.

How does PezT affect cell wall integrity? Other research had suggested that PezT is toxic not only to bacteria but also to yeast. Meinhart's group therefore suspected it might affect a conserved component that is important for both bacterial and yeast survival. One such component is the bacterial cell wall precursor and metabolite UDP-N-acetylglucosamine (UNAG).

In bacteria, UNAG is required for the formation of the peptidoglycans that make up the backbone of the cell wall and give it its stiff structure. The researchers theorized that PezT might act by phosphorylating UNAG because PezT structurally resembles a phosphotransferase. Indeed, their biochemical experiments showed that PezT directly phosphorylates a hydroxyl group on UNAG.

There appear to be many negative consequences of UNAG phosphorylation. For one thing, UNAG is normally the substrate for an enzyme known as MurA. But PezT phosphorylates UNAG at the exact site on which MurA normally acts, thereby preventing UNAG from being processed correctly by MurA. When PezT phosphorylates UNAG, it therefore blocks MurA from acting on an unphosphorylated pool of UNAG. As a result, phosphorylated UNAG gradually accumulates within the cells, aggravating its own blockading effects by a positive feedback loop. As the blockade takes effect, the creation of new cell wall material is prevented, and the cell wall weakens until it can no longer withstand the hydrostatic pressure of the bacterium's contents, causing the bacterium to burst open.

Interestingly, bacteria seem most vulnerable to the effects of PezT when they are growing rapidly; bacteria are more resistant to PezT-mediated death when they are growing slowly or are small in size. Therefore, activation of PezT seems to be a method by which rapidly growing bacteria can instigate a suicide program.

Why would bacteria need to commit suicide? Meinhart and colleagues speculate that the suicide of a few bacteria in a rapidly growing population may promote the release of other toxins that can attack their host's cells or competing bacteria, thereby protecting their own. But now that we know about this secret weapon and its modus operandi, in the future it may be possible to deploy it against pathogenic bacteria by designing drugs that subvert this process. PezT could turn out to be a bacterial Achilles heel.

**Mutschler H, Gebhardt M, Shoeman RL, Meinhart A (2011) A Novel Mechanism of Programmed Cell Death in Bacteria by Toxin–Antitoxin Systems Corrupts Peptidoglycan Synthesis. doi:10.1371/journal.pbio.1001033**

